# Enhanced Precision and Safety in Thermal Ablation: O-Arm Cone Beam Computed Tomography with Magnetic Resonance Imaging Fusion for Spinal Column Tumor Targeting

**DOI:** 10.3390/cancers15245744

**Published:** 2023-12-07

**Authors:** Siran Aslan, Mohammad Walid Al-Smadi, István Kozma, Árpad Viola

**Affiliations:** 1 Department of Neurotraumatology, Semmelweis University, 1081 Budapest, Hungary; drsiran.5@gmail.com; 2 Department of Neurosurgery and Neurotraumatology, Dr. Manninger Jenő National Traumatology Institute, 1081 Budapest, Hungary; smadi996@hotmail.co.uk (M.W.A.-S.); istvan.kozma88@gmail.com (I.K.); 3 Doctoral School of Clinical Medicine, Semmelweis University, 1083 Budapest, Hungary; 4 Department of Operative Techniques and Surgical Research, Faculty of Medicine, University of Debrecen, 4032 Debrecen, Hungary; 5 Department of Neurosurgery, Andras Josa Teaching Hospital, 4400 Nyiregyhaza, Hungary

**Keywords:** percutaneous thermal ablation, image-guided procedures, cone beam computed tomography (CBCT), fusion imaging, thermal ablation techniques, secondary spinal tumors, interventional radiology

## Abstract

**Simple Summary:**

Secondary spinal column tumors are relatively common and can be managed using image-guided percutaneous thermal ablation (IPTA). However, relying on one imaging method can lead to overlooking and underestimating many tumors, resulting in ineffective targeting and treatment. Our study investigates a novel approach for addressing these spinal column tumors, which often cause severe symptoms. We use a combination of CBCT and MRI to precisely guide a minimally invasive thermal ablation procedure. This method ensures that we effectively deliver heat to the tumor, enhancing its accessibility and treatment efficiency. Our promising results show successful tumor coagulation and significant symptom improvement in all four patients. This innovative approach can establish an improved technique for targeting spinal column tumors, ultimately enhancing the treatment outcomes and overall quality of life.

**Abstract:**

Spinal metastatic tumors are common and often cause debilitating symptoms. Image-guided percutaneous thermal ablation (IPTA) has gained significant recognition in managing spinal column tumors due to its exceptional precision and effectiveness. Conventional guidance modalities, including computed tomography, fluoroscopy, and ultrasound, have been important in targeting spinal column tumors while minimizing harm to adjacent critical structures. This study presents a novel approach utilizing a fusion of cone beam computed tomography with magnetic resonance imaging to guide percutaneous thermal ablation for four patients with secondary spinal column tumors. The visual analog scale (VAS) evaluated the procedure effectiveness during an 18-month follow-up. Percutaneous vertebroplasty was performed in two cases, and a thermostat was used during all procedures. Imaging was performed using the Stealth Station navigation system Spine 8 (SSS8) and a 1.5T MRI machine. The fusion of CBCT with MRI allowed for precise tumor localization and guidance for thermal ablation. Initial results indicate successful tumor ablation and symptom reduction, emphasizing the potential of CBCT–MRI fusion in spinal column tumor management. This innovative approach is promising in optimizing therapy for secondary spinal column tumors. Further studies are necessary to validate its efficacy and applicability.

## 1. Introduction

Spinal metastatic tumors frequently develop due to the primary tumor’s spread to the spine through blood or lymphatic metastasis, making them common among advanced malignancies [1]. Vertebral body metastases (VBMs) are widespread and account for 90% of spinal column lesions detected on imaging in oncological patients [2]. The majority of VBM cases, approximately 70%, are found in the thoracic vertebrae. Following this, lumbosacral vertebrae account for 22% of cases, and cervical vertebrae represent 8%. These metastases primarily result from hematogenous spread through Batson’s vertebral venous plexus [3]. These spinal tumors typically present with multiple lesions affecting various segments of the spine [4].

The clinical presentation of VBM is diverse, including symptoms that range from back pain and reduced mobility to metastatic spinal cord compression (MSCC), which can lead to lasting neurological deficits due to vertebral body collapse or fracture [2]. In as many as half of these cases, spinal metastases can lead to persistent pain due to direct tumor infiltration into the bone, pathological fractures, the release of pro-inflammatory cytokines by tumor cells, which enhance osteoclast activity, or the compression of nerve roots and the spinal cord [5,6,7]. Pain and neurological impairment, whether or not accompanied by spinal instability, frequently adversely affect patients’ ability to function independently and their overall quality of life [4,8].

Regarding the diagnosis of these spinal column lesions, open spine procedures are the gold standard and are valued for providing sufficient tissue samples and high diagnostic accuracy and efficiency. However, they come with invasiveness, a tendency to infections, and associated higher morbidity rates [9,10,11,12]. As an alternative, closed biopsy methods, including fine-needle aspiration and image-guided percutaneous spine biopsy, provide alternatives with reduced invasiveness and cost-effectiveness [13,14]. Common imaging modalities for percutaneous spine biopsies include fluoroscopy, ultrasound, CT, and MRI [12,14,15,16,17]. However, improving the accuracy of closed biopsy and cost efficiency to surpass open biopsy remains a critical challenge [18]. Emerging fusion techniques, like CBCT–MRI-guided biopsy, promise to enhance accuracy and efficiency [19]. Further large-scale studies are needed to validate their efficacy.

Among the evolving treatment modalities, image-guided percutaneous thermal ablation (IPTA) stands out for its minimally invasive nature. IPTA directly delivers heat to the tumor, employing techniques like radiofrequency or microwave energy. This method is resource-efficient and reduces morbidity and mortality compared to traditional procedures [20]. IPTA encompasses various techniques, from ethanol ablation and cementoplasty to laser photocoagulation and cryoablation. Each method’s unique advantages and challenges contribute to the broader goal of reducing tumor size, consolidating the spine, and managing the symptoms, particularly the pain [20,21].

Among these techniques, radiofrequency ablation (RFA) has demonstrated efficacy in achieving tumor control. RFA employs high-frequency alternating current to generate localized heat within the tumor, resulting in thermal coagulation and tumor cell destruction. This method is precious for patients with secondary spinal tumors, often challenging due to their location and proximity to critical structures [21,22,23].

IPTA is a promising technique for managing spinal column tumors due to its precision and potential to prevent local tumor progression. Traditional guidance modalities, such as CT, fluoroscopy, and ultrasound, have been crucial in targeting tumors while minimizing damage as much as possible to adjacent critical structures [24,25]. However, with the advancements in imaging technology, fusion techniques that combine multiple modalities are being explored [26]. For instance, the fusion of CBCT with MRI offers the potential for enhanced visualization and precision during the diagnosis of spinal column lesions, and utilizing it would likely be beneficial in the lesion’s ablation [19]. Such fusion techniques have also proven effective in ablating tumors in other regions, like the liver and kidney [27,28,29].

Our goal is to enhance the precision and safety of percutaneous thermal ablation for spinal tumors by utilizing the accurate diagnostic and guidance capabilities of CBCT–MRI fusion. This innovative approach aims to optimize therapeutic outcomes, offering an improved standard of care for individuals with secondary spinal tumors.

## 2. Materials and Methods

### 2.1. Patient Selection and Follow-Up

The study cohort comprised four patients with various spinal tumors selected for surgery. In the patient selection process, specific criteria were applied. These criteria required that the tumor be located within the spinal column without any associated neurological deficits or direct contact with the spinal cord or nerve roots. This precaution was taken to prevent potential heat-related damage to these critical structures. Patients were also required to present with localized pain without radiation to the legs.

Furthermore, patients who had previously undergone radiation therapy for previous tumors, such as prostate cancer, and had reached the maximum allowable radiation dose were considered eligible for inclusion in the study. Pain levels were assessed using the visual analog scale (VAS) before the surgery and at the third, ninth, twelfth, and eighteenth post-operative months. A follow-up MRI was conducted 6 months post-surgery and another at 12 months to monitor for any signs of tumor recurrence. 

### 2.2. Image-Guided Navigation and O-Arm System Setup

We adhered to a specific protocol employing the Stealth Station navigation system Spine 8 (SSS8) (Medtronic Sofamor Danek, Budapest, Hungary) (Video S1). This protocol commenced with the administration of gadolinium contrast-enhanced MRI axial and sagittal scans (1 mm slice thickness) of the spinal segment, utilizing the advanced SIGNA™ Voyager 1.5T MRI machine (GE HealthCare, Budapest, Hungary). After ensuring patient safety and obtaining informed consent, all procedures were conducted under general anesthesia, with patients positioned in the prone orientation on a Maquet carbon operating table. A sterile environment was strictly maintained around the O-arm device (Medtronic Inc., Budapest, Hungary) using the Sterile Tube Drape O-arm System.

To facilitate precise navigation, the Spine Stealth Air reference frame (RF) was securely affixed to the spinous process just above the target vertebral body. The O-arm registration process was achieved within the cranial software integrated into the SSS8 system. Throughout the procedure, the navigation camera consistently ensured clear visibility of both the O-arm trackers and the RF. This continuous integration of the O-arm device with the SSS8 system was established via a robust ethernet cable connection.

### 2.3. Imaging Processing and Analysis

The comprehensive imaging procedure was initiated by acquiring a pre-operative two-dimensional O-arm scans in both axial and sagittal planes (0.83 mm slice thickness), precisely confirming the exact target area. Subsequently, a three-dimensional (3D) scan of the carefully selected spinal region was conducted, encompassing a length of 3–4 levels for the lumbar spine and 4–5 levels for the thoracic spine to ensure thorough coverage. The 3D scan was executed following this verification process by utilizing the state-of-the-art O-arm device. Without delay, these helpful 3D scans were registered and seamlessly transmitted to the SSS8 system, which adhered to the established SSS8 protocol for optimal integration.

A fundamental step was taken within the “Merge Images” menu as the O-arm and MR scans underwent an accurate manual merging process facilitated by utilizing the specialized “Manual Merge” function. The “Verify Merge” option confirmed the seamless merging of the O-arm and MRI scans to ensure these critical scans’ utmost accuracy and alignment. Additionally, the O-arm’s memory function adeptly identified and retained the scan positions and parking configurations of the O-arm device, contributing to the overall precision of the procedure.

Furthermore, the navigating instruments were registered with different colors on the Patient Reference, including essential components such as the Passive Planar Blunt Probe, SureTrak II Large Clamp, and SureTrak II Small Passive Tracker Orange. The SureTrak II Large Clamp was attached to the Johnson and Johnson 8G Vertebroplasty Needle (Johnson and Johnson, Budapest, Hungary). At the same time, the SureTrack II Small Passive Tracker Orange was adeptly linked to the SureTrack II Large Clamp, ensuring thorough coordination and alignment throughout the process.

### 2.4. Biopsy and Coagulation Procedure Workflow

**Figure 1:**
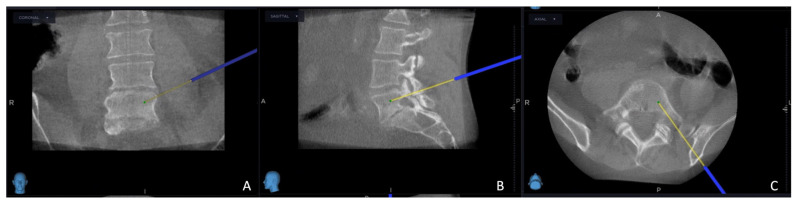
Intraoperative O-arm scan, the yellow projection line shows the entry point: (**A**) coronal view; (**B**) sagittal view; (**C**) axial view.

**Figure 2:**
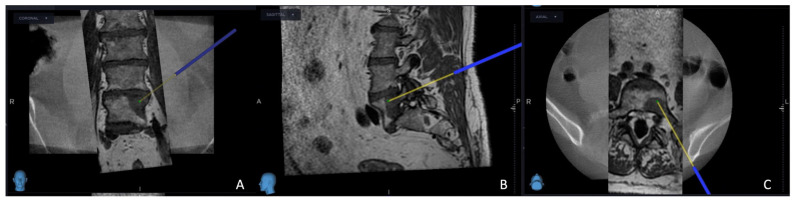
O-arm and MRI fused image, using the MRI image primarily, the yellow projection line, which presents the targeted site for electrode placement: (**A**) coronal view; (**B**) sagittal view; (**C**) axial view.

The intricate procedural sequence embarked upon with the precise determination of the initial entry point was meticulously navigated with the aid of the O-arm scan (Figure 1). Following this pivotal step, the subsequent stage involved the establishment of the targeted point for the upcoming sampling procedure guided by an MRI scan (Figure 2). The surgical roadmap for the needle biopsy procedure was delineated using the instrument projection function. This was followed by a precise 5 mm skin incision, paving the way for the needle biopsy procedure using the Johnson and Johnson 8G Vertebroplasty Needle.

The exact positioning of the needle was methodically verified by cross-referencing its location on both the pre-operative O-arm and MRI scans. Concurrently, the thermostat, a crucial safety component of the procedure, was carefully introduced near the nerve root; additionally, the OsteoCool RF ablation electrode (Medtronic Sofamor Danek, Budapest, Hungary) was carefully positioned, resulting in the establishment of the electrode primer location.

We continued advancing with the needle biopsy at first to obtain a sample of the tumor. After the biopsy, the needle biopsy was removed, and the electrode could be introduced in the same path and method as the needle biopsy was introduced previously; a repeat intra-operative O-arm scan was performed on the same spinal segment, with the main goal of verifying the biopsy’s precise location, ensuring the accuracy of the electrode placement, and confirming the thermostat’s position. This intra-operative O-arm scan was subsequently integrated with the needle biopsy plan selected from the MRI scan using the same previous “Merge Image” function. This fusion was the guiding framework for the precise and safe ablation execution.

**Figure 3:**
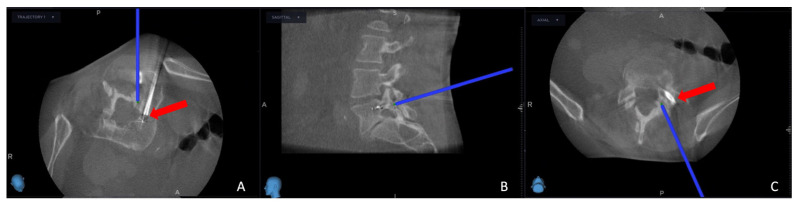
Second intra-operative CBCT scan in the trajectory angled axial view (**A**), sagittal view (**B**), and axial view (**C**). The blue projection line shows the thermostat location, and the red arrow shows the electrode position.

Successful needle biopsy sampling was confirmed if the needle biopsy channel’s position in the second O-arm scan matched the plan selected in the MRI scan, and the sample was sent to the pathology department for tumor typing. Subsequently, the electrode’s and the thermostat’s placement were verified by cross-referencing the registered instrument with the merged image (Figure 3). Thermocoagulation was set at 70 °C and lasted for 7 min, and the process halted upon reaching a thermostat temperature of 39.5 °C. Finally, the electrode, thermostat, and the Spine Stealth Air reference frame were removed. In cases deemed necessary, percutaneous vertebroplasty (PVP) was performed. To carry out the surgeries, a team of two surgeons, one surgical nurse, and one surgery room assistant was required. The duration of surgeries was 90–120 min long, and all patients had a hospital stay of two days, including the surgery day.

## 3. Results

Four patients were enrolled and diagnosed with secondary spinal column tumors in T9, T12, L4, and L5. Among these patients, three had a history of non-Hodgkin lymphoma (two with diffuse large B-cell lymphoma and one with follicular lymphoma), while one had prostate cancer (Table 1). Previously, certain patients underwent cycles of R-CHOP and R-MBACOD, while others received varying doses of radiation therapy and hormone therapy. The duration from the final radiation dose for the primary tumor to the ablation procedure varied between 12 and 33 months, while the time from the diagnosis of these spinal column tumors to the ablation procedures spanned from 12 to 22 days (Table 1). Initially, we conducted PET-CT FDG imaging to detect the lesions. However, due to limited resolution and sensitivity, we subsequently performed MRI investigations for all patients.

**Figure 4:**
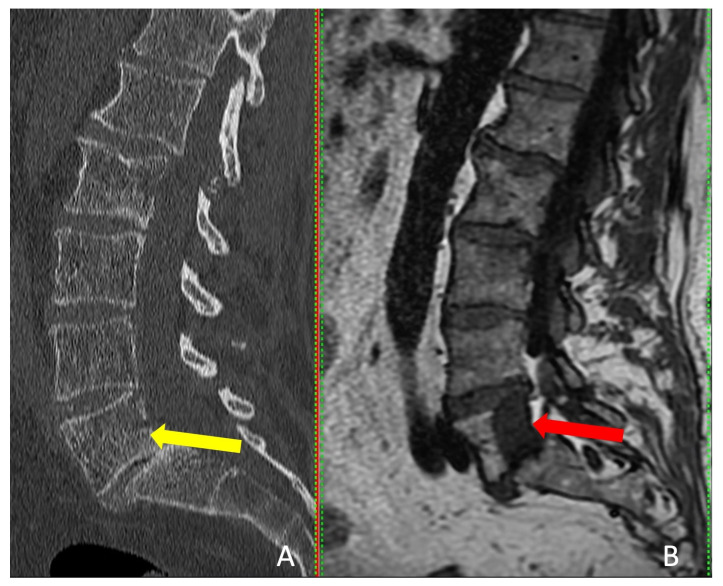
Sagittal view of CT scan (**A**) and MRI scan (**B**). The red arrow indicates the tumor and the yellow arrow shows where the tumor should be detectable.

Interestingly, the tumor was visible on CT in only one patient, but MRI provided more accurate tumor dimensions (Table 2). MRI successfully revealed the tumors in all four patients (Figure 4). These patients presented with localized pain that did not radiate to the legs.

No complications were noted peri-operatively nor in the long term post-operatively. Patients diagnosed with lumbar metastases presented with localized back pain, as indicated by VAS scores of 7/10 and 6/10. However, they reported complete pain relief after three months. Over nine months, twelve months, and eighteen months, the first patient remained pain-free, while the second patient reported VAS scores of 3/10 at nine months and twelve months (Table 3).

Patients with thoracic metastases underwent thermal ablation and percutaneous vertebroplasty (PVP). They reported initial VAS scores of 8/10 and 7/10. After three months, their pain scores decreased significantly to 3/10 and 1/10, respectively. Subsequently, the first patient reported a VAS score 4/10 at nine months and 3/10 at twelve and eighteen months. Conversely, the second patient experienced no pain during the follow-up period (Table 3).

**Figure 5:**
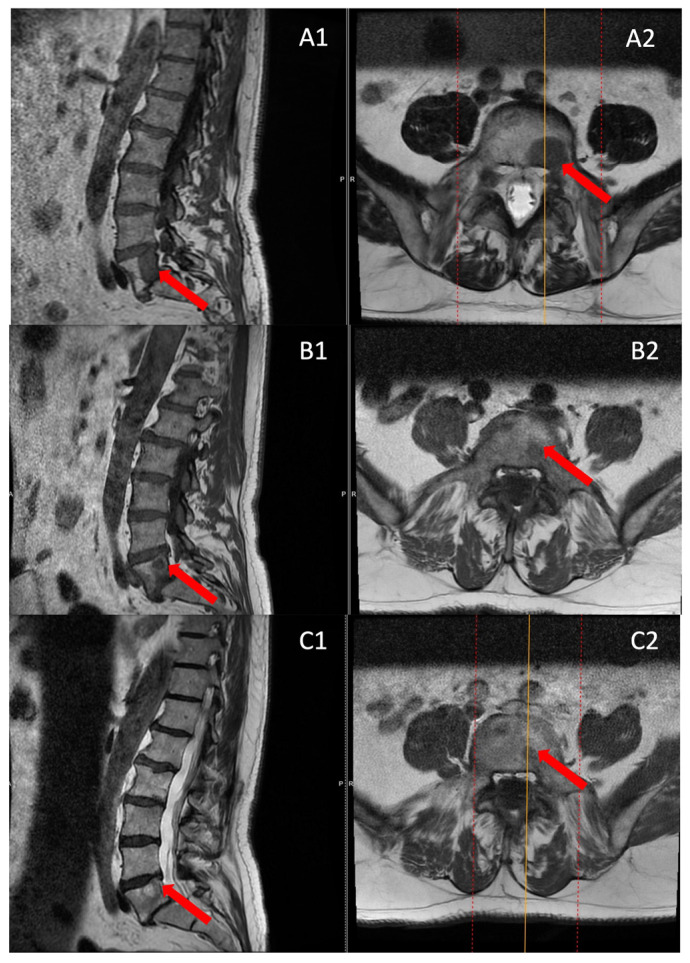
MRI scans before surgery (**A**), 6 months post-surgery (**B**), and 12 months post-surgery (**C**) were examined in both sagittal (A1,B1,C1) and axial sections (A2,B2,C2). The red arrow shows the lesion location.

The follow-up MRI scans conducted at both the 6th and 12th months post-surgery revealed no indications of tumor recurrence in any of the patients, and a reduction in the size of the entire lesion was observed compared to pre-surgery (Figure 5).

## 4. Discussion

This research highlights the critical importance of utilizing a fusion of CBCT and MRI techniques to enhance the effectiveness of percutaneous thermal ablation for spinal column tumors. In a follow-up period of 18 months involving four patients, we observed a general reduction in pain levels, improving their quality of life. The study indicates that ablation guided by fused images can be a viable treatment option for inoperable spinal column tumors resistant to radiation therapy and chemotherapy. Remarkably, no complications or adverse effects were noted in patients throughout the extended follow-up period.

The nerve root and spinal column handled the heat from the radiofrequency ablation procedure without any issues. They did not exceed 39 °C, thanks to the double-checking of electrode placement via a second O-arm scan. While all participants appeared to benefit from the treatment, achieving complete symptom relief for an extended period was impossible in two cases. Additionally, no instances of local tumor recurrence were detected in any of the control MRI post-operatively.

The CBCT–MRI fusion in guiding percutaneous thermal ablation for spinal column tumors inherently emphasizes precision as the utmost consideration. Our procedures ensured precision by engaging in pre-operative discussions and thorough MRI and CT scan analyses. These analyses enabled us to draw potential scenarios using projection lines, providing a comprehensive roadmap for the procedure. Moreover, our careful verification of needle biopsy, electrode, and thermostat placement through cross-referencing with imaging scans, and the intra-operative biopsy results with the integration of an intra-operative O-arm scan with the MRI plan, contributed significantly to maintaining the highest levels of precision.

Furthermore, repeatability is a fundamental aspect of any medical procedure, particularly when considering the potential application of our approach in a broader clinical context. While our study involved a relatively small patient cohort, the steps and protocols described in our methodology were followed consistently throughout all four procedures, with no deviations or additional steps. This adherence to a standardized protocol and the continuous integration of the O-arm with the SSS8 ensured the procedure could be repeated reliably.

Various treatment modalities have been explored to manage spinal column tumors, aiming to alleviate pain, restore or preserve neurological function, and improve the overall quality of life. Traditionally, surgical resection is the most common modality, aiming to remove the tumor mass and alleviate associated symptoms [30]. However, many surgeons of that era recognized that the surgical procedure had limited potential for restoring neurological function. Some even suggested that surgery’s primary objective should shift from rescuing neurological function to providing effective pain management [31,32,33,34]. The surgery’s invasive nature, potential complications, and the tumor’s location can sometimes limit its applicability [30,35].

In cases where the tumor is located close to the spinal canal or spinal cord, surgical intervention may not be a viable option. It is further aggravated by factors such as tumor-related vertebral fractures, loss of bone due to osteoporosis, and comorbidities that alter the likelihood of surgical stabilization of the spine [25]. If left untreated, spinal column tumors can cause severe pain and potentially progress to neurological impairment. RFA has become an effective technique for destroying primary and secondary malignant tumors, especially in organs such as the liver, kidney, brain, pancreas, lung, breast, thyroid, parathyroid, and bone tissue. Percutaneous vertebroplasty can be used in cases of metastatic tumors to strengthen and palliate damaged vertebral bodies [36].

To accurately detect and target tumors to undergo ablation, it is essential to address the limitations of each imaging technique. While CT scans are often recommended for hard-to-reach lesions due to their high-resolution capabilities, which are particularly useful for visualizing the sides of vertebrae and thus minimizing the risk to neural elements [37], MRI excels in soft tissue and bone marrow contrast and certain lesions such as non-Hodgkin’s lymphoma, can only be visualized using MRI [14,38,39]. A study by Al-Smadi et al. involving 18 patients with spinal column tumors revealed that the tumors were either not visible, or their dimensions were underestimated when relying solely on CT scans, as opposed to MRI [19].

It is important to acknowledge the possible risks associated with IPTA, including major complications like tumor seeding, hemorrhage, pneumothorax, and injuries to the bowel and nerves, as well as minor issues such as pain and post-ablation syndrome [40,41,42,43]. However, in our application of the fusion technique within IPTA, we observed none of these complications, even though we have conducted control MRI, and peri-operative vitals were monitored, highlighting the potential effectiveness and safety of our approach.

IPTA hinges on accurately delivering thermal energy to the tumor site. Any deviation can compromise the efficacy of the treatment or, worse, damage critical structures. By employing the fusion technique, clinicians can harness the strengths of both modalities and navigate the intricate spinal anatomy with unparalleled accuracy, ensuring that the tumor is ablated effectively while preserving the spinal cord and nerve roots.

The benefits of image fusion are not confined to spinal interventions. Studies on percutaneous thermal ablation of liver and kidney tumors have shown that fusion techniques improve targeting accuracy, minimize complications, and enhance therapeutic outcomes [27]. Using FI to guide ablation procedures makes the operators more confident because it helps visualize the lesions and nearby structures better, making ablation more accurate, doable, and safe by showing where lesions are and how they relate to nearby organs [44,45].

While our study demonstrates the promising potential of CBCT–MRI fusion in managing spinal column tumors, we acknowledge several limitations that should be considered. Firstly, the patient number in this study was relatively small, limiting our findings’ generalizability. Nonetheless, we benefited from an 18-month follow-up duration, offering valuable and encouraging glimpses into the enduring sustainability and credibility of therapeutic results achieved through the CBCT–MRI percutaneous thermal ablation.

Our research underscores the critical need for innovative approaches in managing spinal column tumors. The fusion of CBCT with MRI offers a unique opportunity to achieve therapeutic goals in a field where accurate tumor targeting is essential. As we strive for minimally invasive yet effective treatments, such fusion techniques hold immense promise in shaping the future of spinal oncology interventions.

## 5. Conclusions

In conclusion, our study presents a novel and promising approach for managing secondary spinal column tumors through the fusion of CBCT with MRI to guide percutaneous thermal ablation. The study cohort consisted of four patients diagnosed with tumors in different spinal regions, demonstrating the feasibility of this technique across various cases. Our findings indicate a general reduction in pain levels among the patients, thus improving their overall quality of life during an 18-month follow-up period.

This innovative approach holds particular significance for patients with inoperable spinal column tumors that are resistant to traditional treatments such as radiation therapy and chemotherapy. Importantly, the procedure exhibited high safety, with no complications or adverse effects observed. Precise temperature control was achieved, with the electrode’s placement monitored through a second O-arm scan, ensuring that temperatures did not exceed 39 °C. Additionally, the absence of local tumor recurrence in post-operative MRI scans further underscores the procedure’s effectiveness.

Traditional surgical resection, while common, has limitations in restoring neurological function and may not be suitable for tumors near the spinal canal or spinal cord. The minimally invasive nature of percutaneous thermal ablation, guided by the fusion of CBCT and MRI, offers a viable alternative for patients who may not be candidates for traditional surgery. By leveraging the strengths of both imaging modalities, clinicians can accurately target and ablate tumors while safeguarding critical spinal structures.

While our study demonstrates the promise of CBCT–MRI fusion in spinal column tumor management, we acknowledge the study’s limitations, particularly the small patient cohort. However, the 18-month follow-up duration provides valuable insights into the long-term sustainability of therapeutic outcomes. In the future, larger-scale studies are warranted further to validate the efficacy and applicability of this innovative approach. As we continue to explore minimally invasive and precise interventions, the fusion of CBCT with MRI holds great potential in shaping the landscape of spinal oncology treatments.

## Figures and Tables

**Table 1:** This table displays the tumor level, primary cancer type, previous treatments, time between the first therapy, diagnosis and ablation, and respective interventions.

Patients # (Tumor Level)	Primary Tumor	Previous Treatment	First Radiation of the Primary Tumors to Ablation (Months)	Diagnosis of Metastatic Spinal Column Tumor to Ablation (Days)	Interventions
1 (Th IX)	DLBCL	6 cycles of R-MBACOD chemotherapy	33	16	Thermocoagulation + PVP
2 (Th XII)	DLBCL	4 cycles of R-CHOP chemotherapy	18	21	Thermocoagulation + PVP
3 (L IV)	PCa	44/2 Gy radiation therapy for the small pelvic field; 54/2 Gy for prostate and seminal vesicles and 78/2 Gy and hormone therapy for prostate	12	22	Thermocoagulation
4 (L V)	FL	5 cycles of R-CHOP chemotherapy	13	12	Thermocoagulation

Abbreviations: diffuse large B-cell lymphoma (DLBCL), prostate carcinoma (PCa), follicular lymphoma (FL), percutaneous vertebroplasty (PVP), number (#), gray (Gy).

**Table 2:** This table presents tumor dimensions in both CT and MRI.

Patient (Tumor Level)	Tumor Dimension (mm)					
	CT			MRI		
	A	S	C	A	S	C
1 (Th IX)	17.5	18.9	9.8	35.4	21.5	19.3
2 (Th XII)	NV			21.6	26.3	10.8
3 (L IV)	NV			34	39.2	21.2
4 (L V)	NV			28.5	29.8	23.2

Abbreviations: axial view (A), sagittal view (S), coronal view (C), millimeter (mm), not visible (NV).

**Table 3:** This table shows VAS scores during the follow-up period for all patients.

Patient # (Tumor Level)	Pre-Surgery VAS	3 Months VAS	9 Months VAS	12 Months VAS	18 Months VAS
1 (Th IX)	8/10	3/10	4/10	3/10	3/10
2 (Th XII)	7/10	1/10	0/10	0/10	0/10
3 (L IV)	6/10	0/10	3/10	3/10	NA
4 (L V)	7/10	0/10	0/10	0/10	0/10

Abbreviations: visual analog scale (VAS), not applicable (NA), number (#).

## Data Availability

All data produced or examined in this study have been included in this published article.

## Supplementary Materials

The following supporting information can be downloaded at: https://1drv.ms/v/s!AluzjWJsn-ZEsn7EqMN7mg60sePe, Video S1: Step-by-Step Guide: CBCT–MRI IPTA for Spinal Column Tumors.
